# The Ventilation of Hospital Wards

**Published:** 1895-05-11

**Authors:** 


### EDITOR'S LETTER-BOX.

[Our correspondents are reminded that prolixity is a great bar to publication, and that brevity of style and conciseness of statement greatly facilitate early insertion.]

### THE VENTILATION OF HOSPITAL WARDS.

THE HON. SYDNEY HOLLAND (chairman of the Poplar Hospital) writes: All new hospitals are now constructed on what you call the "recognised system of fenestration," though where you got that word from I do not know. In your kindly reference to the Poplar Hospital you express a regret that, owing to the necessary position of the bathrooms, this recognised system is not complete; in other words, that there is a space on one side only of each ward of twenty feet without a window. I am, of course, only a layman, but this system to me seems a barbarous one, and, though I suppose I am only exposing my ignorance, I must say that some better way of ventilating a ward ought to be tried than the system of window opposite window down both sides which is the recognised system of fenestration to which you refer. The objections to it are obvious. Every patient has his back, or rather the back of his head, to a dead wall so that reading in bed is not easy to those who have to lie down. Every patient faces the opposite windows, and only those of your readers who have (as I have) had bad concussion of the brain can realise how painful light in one's eyes is under those circumstances. It is disagreeable under any circumstances. Then, wherever there is a window or glass there is a draught. Every patient has a draught therefore on each side of his bed, be the windows as perfect as possible. I am thankful that the exigencies of our alterations to an old building compelled Mr. Plumbe, against his will, to give us twenty feet of bare wall in each ward. I do not feel that because I criticise the present fashion of "fenestration" that I am compelled to invent another and better. It is, undoubtedly, the best at present, though it is, perhaps, overdone. Where it does not exist, as in some of the older London hospitals, the ventilation is certainly defective. But I think hospital architects might consider its disadvantages, even though it be left to a layman to point them out.

*** The introduction of a window on each side of a bed in general wards is admitted to be the best known principle, if not the best possible. Mr. Holland considers the system "barbarous" on three grounds—(although its use has been found to be attended with advantage to the patients, and its neglect with disadvantage). As to the first objection, if there is light enough for the surgeon to examine there is generally ample light for a patient to read with comfort. As to the second, cases in which light opposite the eyes of a patient proves injurious should be treated in separate wards. As to the third, draughts round the patient's head, these are to a great extent avoided if the beds are placed about two feet from the walls, if the air at the bottom of each window is warmed, and if an upward current is given to all air in the wards. Circular wards meet some of these objections, wards hermetically sealed and artificially ventilated might meet others, but the latter of these plans is neither necessary nor desirable in a temperate climate like ours.—ED. *T. H.*

